# Supplementary material for: Hydrological and lock operation conditions associated with paddlefish and bigheaded carp dam passage on a large and small scale in the Upper Mississippi River (Pools 14–18)
Source: PeerJ. 2022 Aug 2;10:e13822. doi: 10.7717/peerj.13822 (PMC9354739; doi:10.7717/peerj.13822)
Supplement: Supplemental Information 1 — Statistics include temperature, hydraulic head, average maximum hydraulic head when dam is at open-river conditions, and open river days provided by the U.S. Army Corps of Engineers (USACE), which were recorded at each respective dam. [file peerj-10-13822-s001.docx]

|  | **Temperature (°C)** | | | **Hydraulic head (m)** | | | **Open river (days)** | | |
| --- | --- | --- | --- | --- | --- | --- | --- | --- | --- |
| Lock & Dam | *Min* | *Max* | *Mean* | *Min* | *Max* | *Mean* | *2017* | *2018* | *2019* |
| 14 | -0.003 | 29.3 | 12.7 | 0.6 | 3.2 | 2.2 | 0 | 8 | 68 |
| 15 | -0.10 | 28.8 | 12.3 | 0.1 | 4.5 | 2.2 | 4 | 25 | 108 |
| 16 | -0.05 | 29.2 | 13.7 | 0.1 | 2.4 | 0.7 | 138 | 163 | 225 |
| 17 | -0.06 | 29.3 | 12.6 | -0.02 | 1.6 | 0.3 | 188 | 226 | 249 |
| 18 | -0.03 | 29.0 | 13.5 | 0.1 | 2.4 | 0.8 | 92 | 130 | 197 |
| 19 | -0.02 | 28.4 | 13.2 | 4.7 | 11.4 | 9.2 | 0 | 0 | 0 |
