# Supplementary material for: Hydrological and lock operation conditions associated with paddlefish and bigheaded carp dam passage on a large and small scale in the Upper Mississippi River (Pools 14–18)
Source: PeerJ. 2022 Aug 2;10:e13822. doi: 10.7717/peerj.13822 (PMC9354739; doi:10.7717/peerj.13822)
Supplement: Supplemental Information 2 [file peerj-10-13822-s002.docx]

| Year Direction | Commercial Tow | Recreational Vessel | Total |
| --- | --- | --- | --- |
| 2017 |  |  |  |
| Upstream | 2271 | 171 | 2442 |
| Downstream | 2255 | 189 | 2444 |
| Total | **4526** | **360** | **4886** |
| 2018 |  |  |  |
| Upstream | 2712 | 157 | 2869 |
| Downstream | 2782 | 205 | 2987 |
| Total | **5494** | **362** | **5856** |
| 2019 |  |  |  |
| Upstream | 1749 | 182 | 1931 |
| Downstream | 1425 | 220 | 1645 |
| Total | **3174** | **402** | **3576** |
| **Total** | **13194** | **1124** | **14318** |
