# Supplementary material for: Hydrological and lock operation conditions associated with paddlefish and bigheaded carp dam passage on a large and small scale in the Upper Mississippi River (Pools 14–18)
Source: PeerJ. 2022 Aug 2;10:e13822. doi: 10.7717/peerj.13822 (PMC9354739; doi:10.7717/peerj.13822)
Supplement: Supplemental Information 3 — The number of residency events (n) and the percentage of RE of each species is separated by season. The residency duration for both species is broken down by number and percentage of RE: ≤ 20 min, > 20 min to ≤ 60 min, > 60 min to ≤ 360 min, and > 360 min. [file peerj-10-13822-s003.docx]

|  | |  |  | **RE (n)** | | | |  | **% RE** | | | | |
| --- | --- | --- | --- | --- | --- | --- | --- | --- | --- | --- | --- | --- | --- |
| **Species** | | **RE (n)** | **% RE** | **≤ 20 min** | **> 20 min ≤ 60 min** | **> 60 min ≤ 360 min** | **> 360 min** |  | **≤ 20 min** | **> 20 min ≤ 60 min** | **> 60 min ≤ 360 min** | | **> 360 min** |
| Bigheaded carp | |  |  |  |  |  |  |  |  |  |  |  | |
|  | Spring | 28 | 21% | 18 | 6 | 3 | 1 |  | 64% | 21% | 11% | 4% | |
|  | Summer | 102 | 77% | 35 | 21 | 39 | 7 |  | 34% | 21% | 38% | 7% | |
|  | Fall | 3 | 2% | 3 | 0 | 0 | 0 |  | 100% | 0% | 0% | 0% | |
| Paddlefish | |  |  |  |  |  |  |  |  |  |  |  | |
|  | Spring | 291 | 54% | 74 | 77 | 102 | 38 |  | 25% | 27% | 35% | 13% | |
|  | Summer | 211 | 40% | 36 | 50 | 110 | 15 |  | 17% | 24% | 52% | 7% | |
|  | Fall | 31 | 6% | 4 | 13 | 12 | 2 |  | 13% | 42% | 39% | 6% | |
