# Supplementary material for: Hydrological and lock operation conditions associated with paddlefish and bigheaded carp dam passage on a large and small scale in the Upper Mississippi River (Pools 14–18)
Source: PeerJ. 2022 Aug 2;10:e13822. doi: 10.7717/peerj.13822 (PMC9354739; doi:10.7717/peerj.13822)
Supplement: Supplemental Information 4 — Respective Akaike’s information criterion (AIC), Δ AIC, and Akaike’s weights (wi) values, for bigheaded carp and paddlefish in the downstream lock approach at Lock and Dam 15 from 2017–2019 are included for the model set. ID is a unique tag number associated with each individual. Definitions of each parameter are located in Table 1. [file peerj-10-13822-s004.docx]

| **Model** | | **AIC** | **ΔAIC** | **w_i_** |
| --- | --- | --- | --- | --- |
| Bigheaded carp | |  |  |  |
|  | Rec.D.n + Temp + Season + (1 \| ID) | 1350.0 | 0.00 | 0.10 |
|  | Rec.D.n + Season + (1 \| ID) | 1351.2 | 1.25 | 0.05 |
|  | Rec.D.n + Temp + (1 \| ID) | 1351.5 | 1.49 | 0.05 |
|  | Rec.U.n + Temp + Season + (1 \| ID) | 1351.6 | 1.63 | 0.04 |
|  | Rec.U.n + Season + (1 \| ID) | 1351.8 | 1.81 | 0.04 |
|  | Barge.D.n + Rec.D.n + Temp + Season +  (1 \| ID) | 1351.8 | 1.82 | 0.04 |
|  | Rec.D.n + Rec.U.n + Temp + Season +  (1 \| ID) | 1351.8 | 1.83 | 0.04 |
| Paddlefish | |  |  |  |
|  | Barge.D.n + Barge.U.n + Rec.D.n + Rec.U.n  + Temp + (1 \| ID) | 6320.1 | 0.00 | 0.16 |
|  | Barge.D.n + Rec.D.n + Hydraulic.head.m +  Temp +(1 \| ID) | 6320.2 | 0.12 | 0.15 |
|  | Barge.D.n + Barge.U.n + Rec.D.n + Rec.U.n + Hydraulic.head.m + Temp + (1 \| ID) | 6320.4 | 0.31 | 0.14 |
