# Supplementary material for: Hydrological and lock operation conditions associated with paddlefish and bigheaded carp dam passage on a large and small scale in the Upper Mississippi River (Pools 14–18)
Source: PeerJ. 2022 Aug 2;10:e13822. doi: 10.7717/peerj.13822 (PMC9354739; doi:10.7717/peerj.13822)
Supplement: Supplemental Information 5 — N represents the number of unique individuals that were present in the downstream lock approach per year. [file peerj-10-13822-s005.docx]

| **Species** | | **2017** | | **2018** | **2019** |
| --- | --- | --- | --- | --- | --- |
| Bigheaded carp | | | N=11 | N=5 | N=3 |
|  | Spring | | 7 | 1 | 5 |
|  | Summer | | 25 | 11 | 23 |
|  | Fall | | 1 | 1 | 0 |
| Paddlefish | | | N=0 | N=11 | N=38 |
|  | Spring | | 0 | 3 | 56 |
|  | Summer | | 0 | 28 | 36 |
|  | Fall | | 0 | 12 | 2 |
