# Supplementary material for: Hydrological and lock operation conditions associated with paddlefish and bigheaded carp dam passage on a large and small scale in the Upper Mississippi River (Pools 14–18)
Source: PeerJ. 2022 Aug 2;10:e13822. doi: 10.7717/peerj.13822 (PMC9354739; doi:10.7717/peerj.13822)
Supplement: Supplemental Information 6 — Akaike’s information criterion (AIC), Δ AIC, and Akaike’s weights (wi), and area under the receiver operating characteristic curve (AUC) for the confidence set of presence events generalized linear models for bigheaded carp and paddlefish in the downstream lock approach at LD 15 from 2017–2019. AUC.test is the performance of the top model when tested with a 20% subset of original data. Cohen’s kappa (κ) is the accuracy of the prediction to the actual occurrence (<0.00 =poor agreement, 0.00–0.20 = slight agreement, 0.21–0.40 = fair agreement, 0.41–0.60 = moderate agreement, 0.61–0.80 = substantial agreement; Landis and Koch 1977). Definitions of each parameter are located in Table 1. [file peerj-10-13822-s006.docx]

| **Model** | | **AIC_i_** | **ΔAIC** | **w_i_** | **AUC** | **AUC.test** | **κ** |
| --- | --- | --- | --- | --- | --- | --- | --- |
| Bigheaded carp | |  |  |  |  |  |  |
|  | Barge.U.n + Rec.U.n + Hydraulic.head.m + Temp + Year | 312.1 | 0 | 0.24 | 0.90 | 0.89 | 0.43 |
|  | Temp + Hydraulic.head.m + Barge.D.n + Barge.U.n + Rec.D.n + Rec.U.n + Year + Season | 312.8 | 0.68 | 0.17 | 0.90 | 0.91 | 0.45 |
|  | Rec.U.n + Hydraulic.head.m + Temp + Year | 313.3 | 1.15 | 0.13 | 0.89 | 0.89 | 0.38 |
|  | Barge.D.n + Barge.U.n + Rec.D.n + Rec.U.n + Hydraulic.head.m + Temp + Year | 314.1 | 1.94 | 0.09 | 0.90 | 0.89 | 0.43 |
| Paddlefish | |  |  |  |  |  |  |
|  | Temp + Hydraulic.head.m + Barge.D.n + Barge.U.n + Rec.D.n + Rec.U.n + Year + Season | 372.5 | 0 | 0.99 | 0.94 | 0.93 | 0.64 |
