# Supplementary material for: Hydrological and lock operation conditions associated with paddlefish and bigheaded carp dam passage on a large and small scale in the Upper Mississippi River (Pools 14–18)
Source: PeerJ. 2022 Aug 2;10:e13822. doi: 10.7717/peerj.13822 (PMC9354739; doi:10.7717/peerj.13822)
Supplement: Supplemental Information 7 — The orange dots depict the location of a test tag at the time of detection on a receiver. The average detection percentages were calculated for each zone, where the upstream lock approach had two receivers, the lock chamber had two receivers, and the downstream lock approach included 11 receivers. [file peerj-10-13822-s007.pdf]

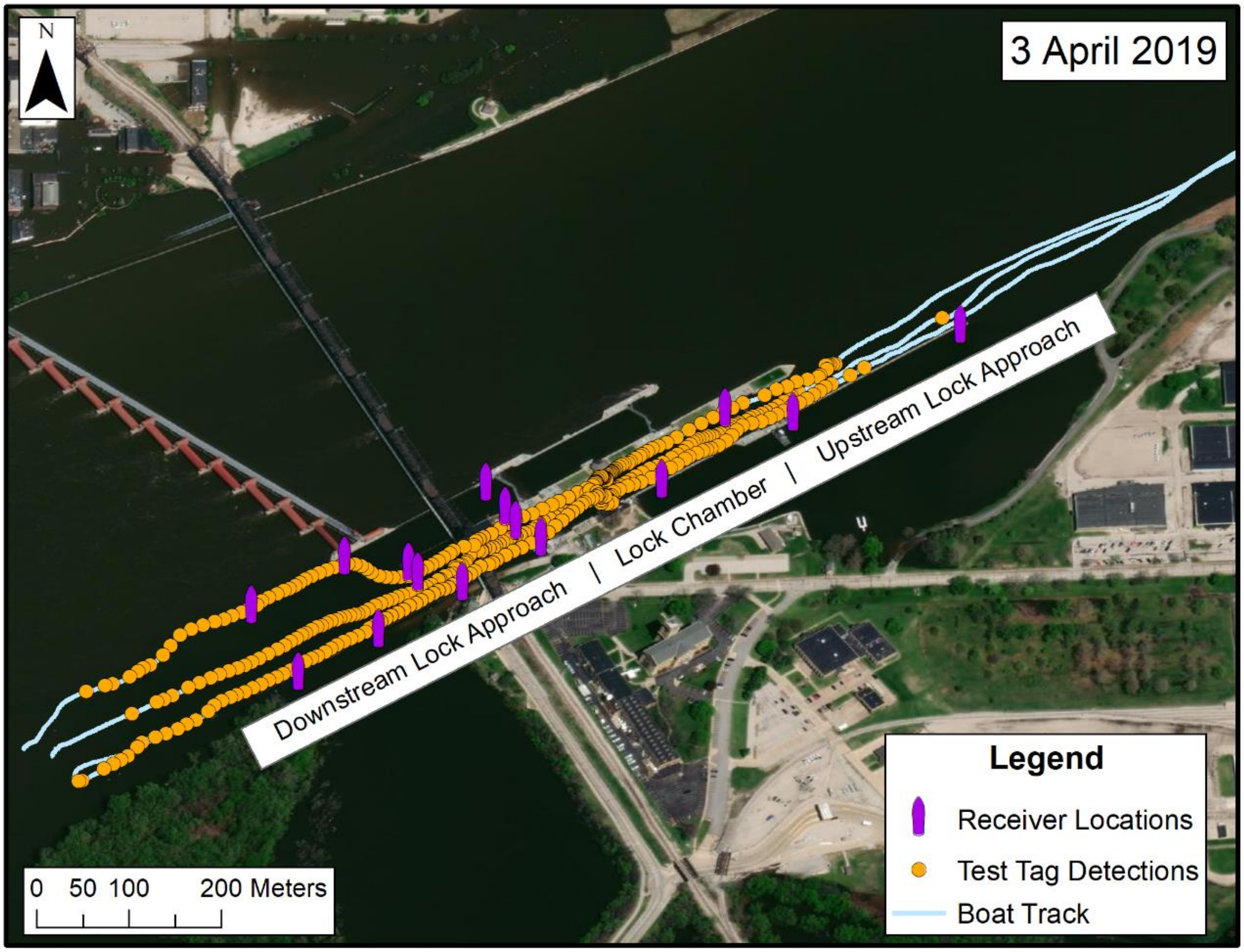

Service Layer Credits: Source: Esri, Maxar, GeoEye, Earthstar Geographics, CNES/Airbus DS, USDA, USGS, AeroGRID, IGN, and the GIS User Community
